# Relationship between Isometric Muscle Force and Fractal Dimension of Surface Electromyogram

**DOI:** 10.1155/2018/5373846

**Published:** 2018-03-15

**Authors:** Matteo Beretta-Piccoli, Gennaro Boccia, Tessa Ponti, Ron Clijsen, Marco Barbero, Corrado Cescon

**Affiliations:** ^1^Rehabilitation Research Laboratory 2rLab, Department of Business Economics, Health and Social Care, University of Applied Sciences and Arts of Southern Switzerland, Manno, Switzerland; ^2^Criams-Sport Medicine Centre Voghera, University of Pavia, Pavia, Italy; ^3^NeuroMuscular Function Research Group, School of Exercise and Sport Sciences, Department of Medical Sciences, University of Turin, Turin, Italy; ^4^CeRiSM (Research Centre for Sport, Mountain, and Health), Rovereto, Italy; ^5^University of Business Economics, Health and Social Care, University of Applied Sciences and Arts of Southern Switzerland, Landquart, Switzerland

## Abstract

The relationship between fractal dimension of the surface electromyogram (sEMG) and the intensity of muscle contraction is still controversial in simulated and experimental conditions. To support the use of fractal analysis to investigate myoelectric fatigue, it is crucial to establish the interdependence between fractal dimension and muscle contraction intensity. We analyzed the behavior of fractal dimension, conduction velocity, mean frequency, and average rectified value in twenty-eight volunteers at nine levels of isometric force. sEMG was obtained using bidimensional arrays in the biceps brachii muscle. The values of fractal dimension and mean frequency increased with force unless a plateau was reached at 30% maximal voluntary contraction. Overall, our findings suggest that, above a certain level of force, the use of fractal dimension to evaluate the myoelectric manifestations of fatigue may be considered, regardless of muscle contraction intensity.

## 1. Introduction

The relation between electromyography (EMG) and force has been a controversial topic for more than four decades. The surface EMG (sEMG)/force relationship strongly depends on motor units (MUs) control by the central nervous system (CNS) and by the peripheral features of muscle. The CNS modulates the force expressed by the muscle by controlling two parameters: the recruitment of MUs and the firing rate of active MUs [1]. These two parameters are directly connected with the generation of electrical activity inside the muscle and also influence the sEMG signal [2]. Indeed, the sEMG signal is a result of the interferential summation of MU action potentials (MUAPs) detected by electrodes and thus it is of interest to understand the role played by the neural parameters in driving the sEMG-force relationship [3]. The shape of this relationship has been explored in experimental and simulation studies, with conflicting results ranging from linearity to nonlinearity [4–11]. The shape of this relationship might also depend on the muscle investigated, muscle fiber composition, and muscle fiber size [9, 12].

Inconsistent results in the literature may also reflect that muscles are not necessarily uniformly activated at increased loads in a specific action. For this reason, sEMG varies spatially over the muscle belly [13–15]. Applying multichannel array electrode systems in sEMG recordings has been demonstrated to improve the extraction of reliable sEMG/force relationship increasing the representability of the measured sEMG signal [16–18].

Great interest has been given in the literature to the nonlinear feature of the sEMG signal, such as recurrent quantification analysis, percentage of determinism, sample entropy, normalized mutual information, and fractal dimension (FD) [19–23]. Nonlinear analysis offers a powerful approach for the investigation of physiological time series because it provides a measure of the signal complexity. In particular, the FD of the signal is a measure of self-similarity over multiple time scales. Several studies [23–27] have applied box-counting methods to estimate the FD of the sEMG signal and a recent investigation showed a good reliability of FD during isometric contractions in the biceps brachii muscle [28].

Nonlinear feature of the sEMG has been widely applied to monitor the myoelectric manifestations of fatigue during the course of isometric contractions [29]. Indeed, during sustained submaximal contractions, the alterations in the activity of muscles undergoing fatigue can be quantified, using linear or nonlinear methods, prior to task failure [29]. Mesin and colleagues [30] computed a combination of both linear and nonlinear analysis to synthetic and experimental sEMG signals. They found that FD was the most related to the level of synchronization and the least related to the changes of muscle fiber conduction velocity (CV). Consequently, they proposed the combination of FD and CV as bidimensional index providing information about the central and peripheral adjustments occurring during fatigue [30]. In a more recent simulation study, Mesin and colleagues [31] found that beyond synchronization level, the FD of the EMG signals increased with the average firing rate of the active MUs. For this reason, recently, the combined monitoring of muscle fiber conduction velocity (CV) and FD parameters during continuous contractions was applied in the evaluation of myoelectric manifestations of fatigue [32–34]. However, to fully understand the applicability of FD analysis in the study of myoelectric manifestations of fatigue, it is crucial to determine if the FD is also affected by the level of force exerted by muscles.

Some studies found that the FD of sEMG was linearly but weakly related to the contraction level (% of maximal voluntary contraction, MVC) in simulated and experimental conditions [23, 25, 26]. However, recent investigations showed that FD is not related to the intensity of muscle contraction [35, 36]; therefore, the relationship between force and the FD of sEMG is still controversial. Thus, the aim of this study was to evaluate the relationship between force and FD of sEMG during isometric contractions of the biceps brachii.

## 2. Materials and Methods

### 2.1. Participants

The study was approved by the local ethics committee of the Swiss Italian Health and Sociality Department, Switzerland. All procedures were conducted according to the Declaration of Helsinki. All participants signed a written informed consent form before participation in the experiments. Twenty-eight healthy recreationally active volunteers (14 women and 14 men) aged between 20 and 36 years (25 ± 4 yrs) from a university setting were recruited to participate in the study.

### 2.2. Experimental Procedure

The subjects participated in three experimental sessions (“trials 1–3”): the first two trials were conducted within the same day, with four minutes of rest in between, without repositioning the electrodes. The third trial was performed a week apart under the same environmental conditions.

**Figure 1:**
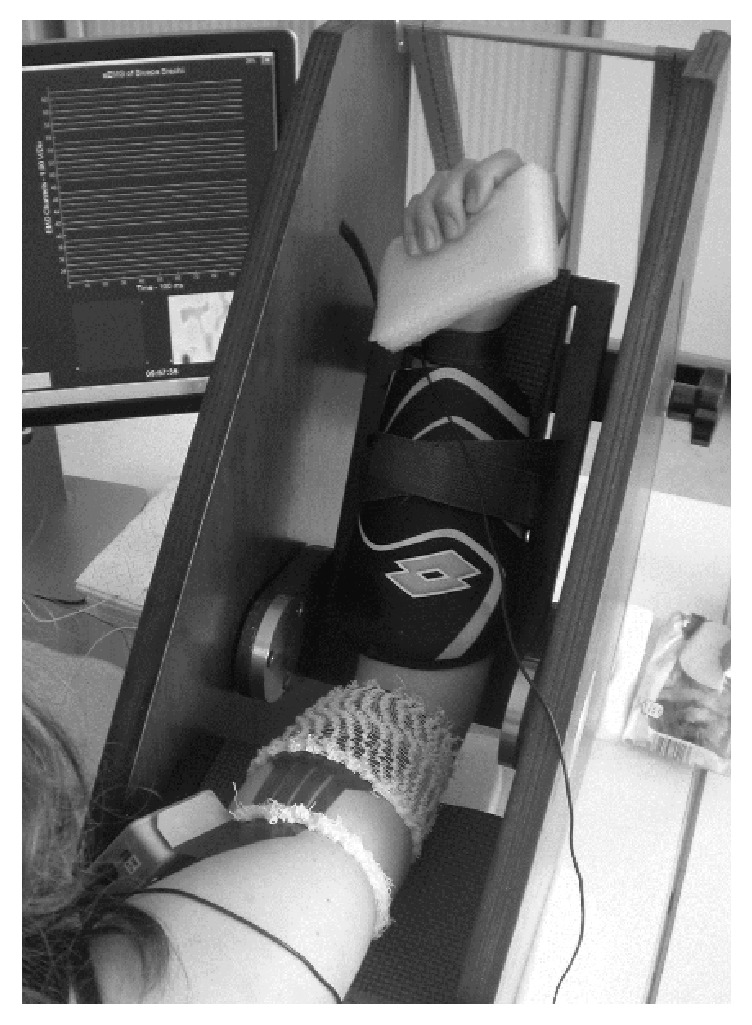
Electrode array position on biceps brachii muscle.

Subjects were seated in a height-adjustable chair with their arm positioned on an isometric ergometer (MUC1, OT Bioelettronica, Turin, Italy), equipped with a load cell (Model TF022, CCT Transducers, Turin, Italy). In order to isolate the action of the biceps brachii, the wrist was fastened to the ergometer, with the elbow at 120°, as shown in Figure 1.

Initially, two isometric MVCs were performed, separated by a 2-minute rest. During each contraction of the trial, the force trace was displayed to participants on a computer monitor as visual feedback. Participants were instructed to increase the force up to their maximum and to hold it for 2-3 s. Participants were given strong verbal encouragement.

Next, after a 4-minute rest, the subjects performed a sequence of nine short contractions, from 10 to 90% of their MVC in steps of 10% MVC in randomized order, lasting 5 s, with 20 s of rest in between. After each contraction, the subjects were asked to provide a value of the perceived exertion on a visual Borg scale, ranging from 6 to 20 [37]. In the first day of measurement, after the first session (trial 1), a second sequence of contraction, constituting trial 2, was performed.

### 2.3. EMG and Force Measurements

Myoelectric signals were detected from the biceps brachii, in a monopolar configuration using a bidimensional array of 64 electrodes (3 mm diameter, 8 × 8 grid, and 10 mm interelectrode distance; model ELSCH064NM3; OT Bioelettronica) (Figure 1). This muscle was chosen in order to obtain high-quality sEMG signals according to the qualitative criteria described in [38]. The electrode grid was applied on the muscle belly, with its distal edge close to the cubital fossa and the midline of the array aligned with the midline of biceps along a line from the cubital fossa to the acromion (see Figure 1). A ground electrode was placed on the contralateral wrist. The EMG signals were amplified (EMG-USB2; OT Bioelettronica), band-pass filtered (10–750 Hz), sampled at 2048 Hz, and stored on a computer.

The isometric ergometer was used to measure elbow torque with a torque meter operating linearly in the range 0–1000 Nm. The torque signal was amplified (MISO II; OT Bioelettronica) and stored on a computer with the sEMG data. The torque signal was displayed on a screen, providing real-time biofeedback.

### 2.4. Signal Processing

The number of channels used for CV estimation was selected based on visual inspection of single differential signals, between the distal tendon and the innervation zone, along one of the array columns, as previously described [28].

The number of channels chosen to estimate CV was between 4 and 7, according to a previously published study [39]. CV values outside the physiological range (3–6.5 m/s) were excluded from the analysis [40].

For each signal, a 3 s lapse was identified, where the force level was stable within the 10% boundaries of the target force requested to the subjects. Signals were then divided into epochs of 1 s and CV was computed using a multichannel algorithm [41] on the selected channels. The three obtained values were then averaged. Next, each of the three epochs of each signal was used for the estimation of average rectified value (ARV), mean frequency of the power spectrum (MNF), and FD. Estimates obtained from single channels were averaged over the channels previously selected by visual analysis and over the three signal epochs. Therefore, for each contraction level one value for ARV, MNF, and FD was obtained.

In addition, ARV, MNF, CV, and FD data, as well as Borg scale values, were normalized for each subject according to their values at 70% MVC and expressed as percentages. The force level of 70% was selected after the completion of data collection, since many of the subjects could not perform 80 and 90% MVC contraction. The 70% value was the maximum force level which all the subjects could reach.

FD was estimated using the box-counting method, as previously reported [28]. Data were analyzed by custom-written software in MATLAB R2014b (Mathworks, Natick, USA)

### 2.5. Statistical Analysis

Intra- and intersession reliability were examined using the Intraclass Correlation Coefficient (ICC_(2,1)_) on averaged measures [42], since its use has been recommended in reliability studies [43, 44]. The criteria used for the interpretation of the ICCs were as follows: 0.00–0.25: no correlation; 0.26–0.49: low correlation; 0.50–0.69: moderate correlation; 0.70–0.89: high correlation; 0.90–1.00: very high correlation [45].

To test the relationship between EMG variables and force, only the first session, that is, trial 1, was considered. A Shapiro–Wilk test revealed that all the estimated EMG variables were not normally distributed across subjects and, thus, the nonparametric Kruskal–Wallis test was performed on the sEMG variables for each contraction at difference force levels. Considered factors were trial and force level. When the Kruskal–Wallis test indicated significant variations, a post hoc Dunn–Bonferroni test [46] was applied on pairwise comparisons; statistical significance was accepted at the *p* < 0.001 level.

The epsilon-squared estimate of effect size was calculated using [47]

(1) $$\begin{array}{c} E_{R}^{2}=\frac{H}{n-1}, \end{array}$$

where *H* is the value obtained in the Kruskal–Wallis test (the K-W* H*-test statistics) and *n* the total number of observations. The *E*_*R*_^2^ coefficient assumes values between 0 (indicating no relationship) and 1 (perfect relationship).

Statistical analyses were performed using SPSS version 22.0 (SPSS Inc., Chicago, IL, USA), and significance was set to *α* = 0.05. Results are reported as median and interquartile range.

## 3. Results

### 3.1. Reliability Analysis

Table 1 documents the results of ICC_(2,1)_ analysis for the initial values of CV, FD, MNF, and ARV during the short isometric contractions, with force levels between 10 and 90% MVC. According to the classification of [45], high to very high levels of intrasession reliability were identified for all the parameters (ICC between 0.86 and 0.97), whereas the intersession reliability was considerably lower. The most reliable parameter across experimental sessions was indeed ARV, followed by FD and MNF. Initial values of CV showed higher ICC values at lower contraction levels, whereas at force levels between 70% and 90% MVC, CV displayed a very low intersubject variability, demonstrating dependence on days and trials larger than dependence on subjects [39, 48].

### 3.2. Relation with Force

**Figure 2:**
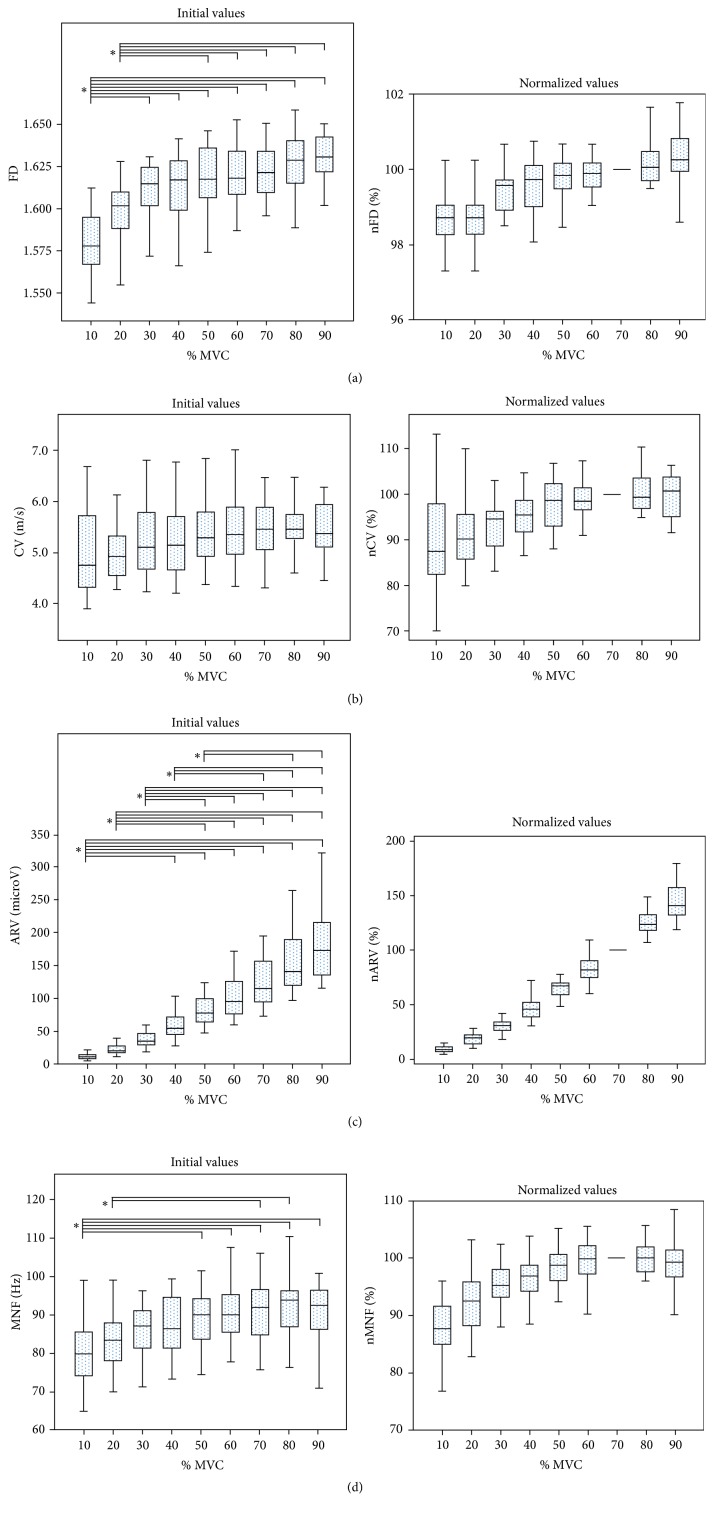
Box-and-whisker plots of initial and normalized values (with respect to their values at 70% MVC) of fractal dimension (FD) conduction velocity (CV), average rectified value (ARV), and mean frequency (MNF) during short isometric 10–90% maximal voluntary contractions (MVCs) of the biceps brachii. Statistically significant results of the Dunn–Bonferroni post hoc test are indicated (^*∗*^*p* < 0.001).

Kruskal–Wallis test did not reveal any statistical dependence of the variables on trials. Distributions of FD, ARV, MNF, and Borg ratings were similar for all contraction levels, as assessed by visual inspection of boxplots (Figure 2). Median scores of these parameters were statistically different across the nine levels of force (*p* < 0.0001). Only the increasing trend of CV versus force was not statistically significant; for this reason no post hoc analysis was performed for CV.

**Figure 3:**
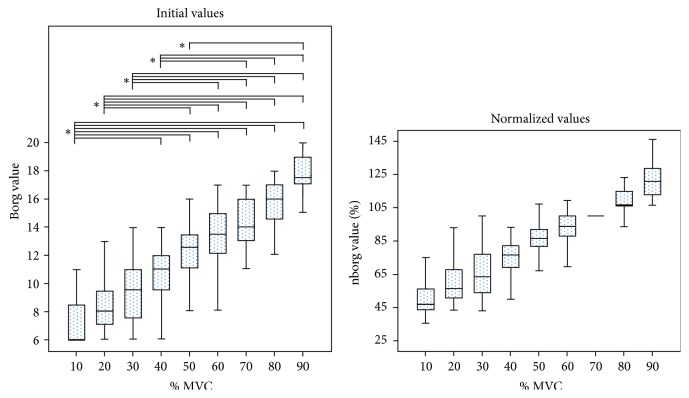
Box-and-whisker plots of the initial and normalized values (with respect to their values at 70% MVC) of Borg ratings during short isometric 10–90% maximal voluntary contractions (MVCs) of the biceps brachii. Statistically significant results of the Dunn–Bonferroni post hoc test are indicated (^*∗*^*p* < 0.001).

To allow better visualization of the parameters trend, a boxplot for each normalized parameter with respect to their values at 70% MVC was added to Figure 2. Effect size analysis, that is, the percentage of the variability of the considered parameters which is really accounted for by the level of force, revealed very high scores for ARV and Borg values (epsilon-squared estimates, resp., 87% and 70%), whereas smaller effect size was found for FD and MNF (epsilon-squared estimates, resp., 37% and 17%). The post hoc analysis revealed statistically significant differences in the considered parameters obtained at low force levels (resp., 10–40% MVC for ARV and Borg ratings and 10–30% MVC for FD and MNF) with respect to high force levels (50–90% MVC) (Figures 2 and 3).

## 4. Discussion

### 4.1. Intra- and Intersession Reliability

FD, MNF, and ARV showed high intra- and intersession reliability, in accordance with previously published studies [28, 39, 48–51]; the intersession reliability of CV at contraction levels higher than 60% MVC was very low. This result might be explained by the fact that the variability of CV between subjects decreases as the level of contraction increases over 60% MVC [52].

### 4.2. Relation between EMG Parameters, Borg Ratings, and Force

In the present study, FD and MNF were the variables least influenced by the level of exerted force (Figures 2(a) and 2(d)). In fact, both variables showed a trend, increasing from 10% to 30% MVC, but thereafter reaching a plateau beyond 30% of MVC (confirmed by the results of the post hoc analysis, as well). The little or even independence of FD and MNF on the level of muscle force was reported also in two previous investigations in other muscles and with different methods [36, 53]. In particular, in [36] the upper trapezius muscle was investigated, which was compared to the biceps brachii, and presents a much more complex architecture and an heterogeneous distribution of the muscle activity [54].

As already reported in literature, FD is sensitive to the presence of large active MUAPs that usually appear in the signal due to synchronization at high force levels, during fatiguing contractions [30]. Nevertheless, a similar phenomenon happens also at low force levels, whenever larger MUs, with low firing frequency, are recruited according to the Henneman's size principle. Moreover, in simulated EMG signals, FD was positively correlated to the firing rate of the active MUs and negatively correlated to the level of MU synchronization [31]. Since the level of synchronization is not expected to change in nonfatiguing contractions, it was reasonable to hypothesize that FD could somehow increase with increasing force levels. Thus, it is possible to speculate that FD might be a reliable indicator of MU synchronization, less dependent from the firing rate.

Muscle fiber CV seems to be the most affordable variable for relating EMG signals modifications and MUs pool recruitment [55]. Since CV increases gradually when larger MUs are recruited [56], it was expected to increase with contraction intensity [40]. Contrary to the expectation, the average CV did not increase significantly with increasing force levels although we could observe a trend in that direction in our dataset (see Figure 2(b)). There are two main confounding factors that could have affected CV estimates: (1) the subcutaneous tissue and (2) the alignment of the electrode grid along the direction of muscle fibers. Indeed, a high thickness of subcutaneous tissue and malalignment of electrode grids might both produce an overestimation of CV and consequently affect the trend of CV across force levels. Since the CV values were relatively high (>4.5 m/s) even at the lowest force levels (i.e., 10% of MVC), this explanation seems to be plausible. Anyway, an overestimation of CV, if present, would be visible at all contraction levels; thus normalized values would not be affected by this bias.

The amplitude of the EMG signal (ARV) was the variable most dependent on the level of force exerted (Figure 2(c)). This was an expected result, since many previous studies demonstrated a direct relationship between EMG and force [4–11]. In particular, ARV values obtained at the highest force, that is, the 90% of the MVC, were greater than those lower or equal to the 50% of MVC. Whereas, between 60% and 90% of MVC, no increase in ARV was found. Thus, EMG amplitude seemed to be sensitive to the increase of force only from low (10% of MVC) to medium (50% of MVC) force levels, but not from 50% to 90%. Even this was an expected result because Troiano and colleagues previously reported the same pattern [36]. The recruitment of motor units and the firing rate of active motor units progressively increase at increasing force exertion [1], and this leads to increasing electrical activity inside the muscle [2]. Consequently, increasing amplitude of EMG signal would be expected throughout the whole range of forces. However, our results showed that the EMG amplitude was not consistently affected by the increase in force after 50% of MVC. This can be explained by the fact that the amplitude cancellation influenced the measures of EMG amplitude mostly at high force levels. Indeed, the amplitude cancellation has been proven to increase with increasing number of active motor units [57].

Finally, the present study found a relation between ratings of perceived exertion (Borg ratings) and force levels (Figure 3) in-line with previous published studies, where a linear relationship, during isometric contractions, was found [36, 58, 59]. Interestingly, as occurred with ARV, no statistically significant increase in perceived exertion was found between 60% and 90% of MVC. Together, these results furthermore support previous findings indicating the relationship between muscle activation and perceived exertion [60].

The limitations of this study are mainly related to technical constraints. Firstly, we investigated only one muscle, which, of course, does not represent the behavior of all the muscles. Secondly, to our knowledge, literature is currently lacking studies on validity of FD in estimating MU synchronization. If future studies will overcome this gap, FD will provide a valid and robust measure of MU synchronization during fatiguing contractions.

## 5. Conclusions

The present study showed that FD is a reliable EMG parameter at all contraction levels and has little dependency from muscle force, in the biceps brachii muscle above 30% MVC. In such conditions, FD can be applied in experimental studies focusing on fatigue or on motor unit synchronization, independently from the force exerted.

## Figures and Tables

**Table 1:** Results of the reliability analysis of initial values of CV, FD, MNF, and ARV at 10 to 90% MVC. Intra- and intersession ICC scores are reported.

MVC	ICC		ICC		ICC		ICC	
	Intra	Inter	Intra	Inter	Intra	Inter	Intra	Inter
	CV		FD		MNF		ARV	
10%	0.95	0.79	0.86	0.74	0.94	0.76	0.92	0.83
20%	0.97	0.76	0.86	0.78	0.96	0.86	0.86	0.74
30%	0.98	0.77	0.91	0.81	0.97	0.78	0.96	0.87
40%	0.97	0.68	0.94	0.85	0.97	0.75	0.90	0.85
50%	0.96	0.39	0.91	0.81	0.97	0.59	0.88	0.83
60%	0.96	0.59	0.89	0.90	0.96	0.79	0.89	0.81
70%	0.90	0.21	0.87	0.70	0.96	0.77	0.96	0.87
80%	0.91	0.04	0.94	0.82	0.96	0.73	0.96	0.89
90%	0.96	0.22	0.90	0.81	0.96	0.76	0.96	0.81

*Note*. MVC: maximal voluntary contraction; ICC: intraclass correlation coefficient intra- and intersession.
